# Emicizumab for acquired hemophilia A: Report of two cases and dosing strategies

**DOI:** 10.1002/jha2.878

**Published:** 2024-03-15

**Authors:** Faiza Ahmed, Mariia Kasianchyk, Alejandro Moreno, Simone Chang, Satish Maharaj

**Affiliations:** ^1^ Hematology and Oncology, Department of Internal Medicine Texas Tech University El Paso Texas USA

**Keywords:** acquired, dosing, emicizumab, hemophilia, inhibitor

## Abstract

Acquired hemophilia A (AHA) is a rare autoimmune bleeding disorder caused by autoantibodies against FVIII. Severe AHA is life‐threatening. Currently, licensed hemostatic agents for the treatment of severe AHA have short half‐lives and require intravenous administration, leading to a need for hospitalization, higher costs, and negative effects on quality of life. We present two cases of severe AHA with high inhibitor titers where emicizumab was safely and effectively used with intensive immunosuppression. These reports suggest in vivo efficacy even in high inhibitor environments. The optimal dosing regimen (accelerated vs. standard loading, maintenance frequency) is unknown and we discuss the current approaches.

## 1 INTRODUCTION

Acquired hemophilia A (AHA) is a rare acquired bleeding disorder involving the generation of autoantibodies against FVIII [1]. AHA is not inherited and incidence increases with age; estimated at 1–2 cases per million [2]. Patients often present with spontaneous or provoked bleeding that is cutaneous, intramuscular, or retroperitoneal in location [3]. Following stabilization of initial hemorrhage, rebleeding occurs in approximately 50% of patients and this risk remains significant until near‐normal FVIII levels are attained [4]. Hemorrhage in AHA can be limb‐, organ‐, or life‐threatening [5].

Standard therapy includes 1) immunosuppressive therapy (corticosteroids, rituximab, and cyclophosphamide) to suppress inhibitor formation and 2) agents for hemostasis such as recombinant activated human Factor VII (rFVIIa), activated prothrombin complex concentrate (aPCC), and recombinant porcine FVIII concentrate (rpFVIII) [6, 7]. These treatments are limited by short half‐lives and intravenous route of administration. Both rFVIIa and aPCC require hospitalization, leading to increased cost and decreased quality of life. Some patients with AHA have cross‐reactive antibodies to rpFVIII, while those who do not, often develop anti‐FVIII alloantibodies with repeated rpFVIII exposure [8].

Emicizumab is a humanized, recombinant antibody with bispecific properties that mimic the role of coagulation FVIII [9]. While emicizumab has been Food and Drug Administration‐licensed for use in bleeding prevention for patients with congenital Hemophilia A (CHA) with and without inhibitors, it has not been approved for use in AHA. There are emerging reports of patients with AHA who show significant clinical improvement on emicizumab; optimal dosing and activity remain unknown, especially in severe AHA [10]. Here, we discuss two cases of severe AHA effectively managed with emicizumab treatment without any adverse events and complete remission without rebleeding.

## 2 CASE REPORTS

### 2.1 Case 1

**FIGURE 1:**
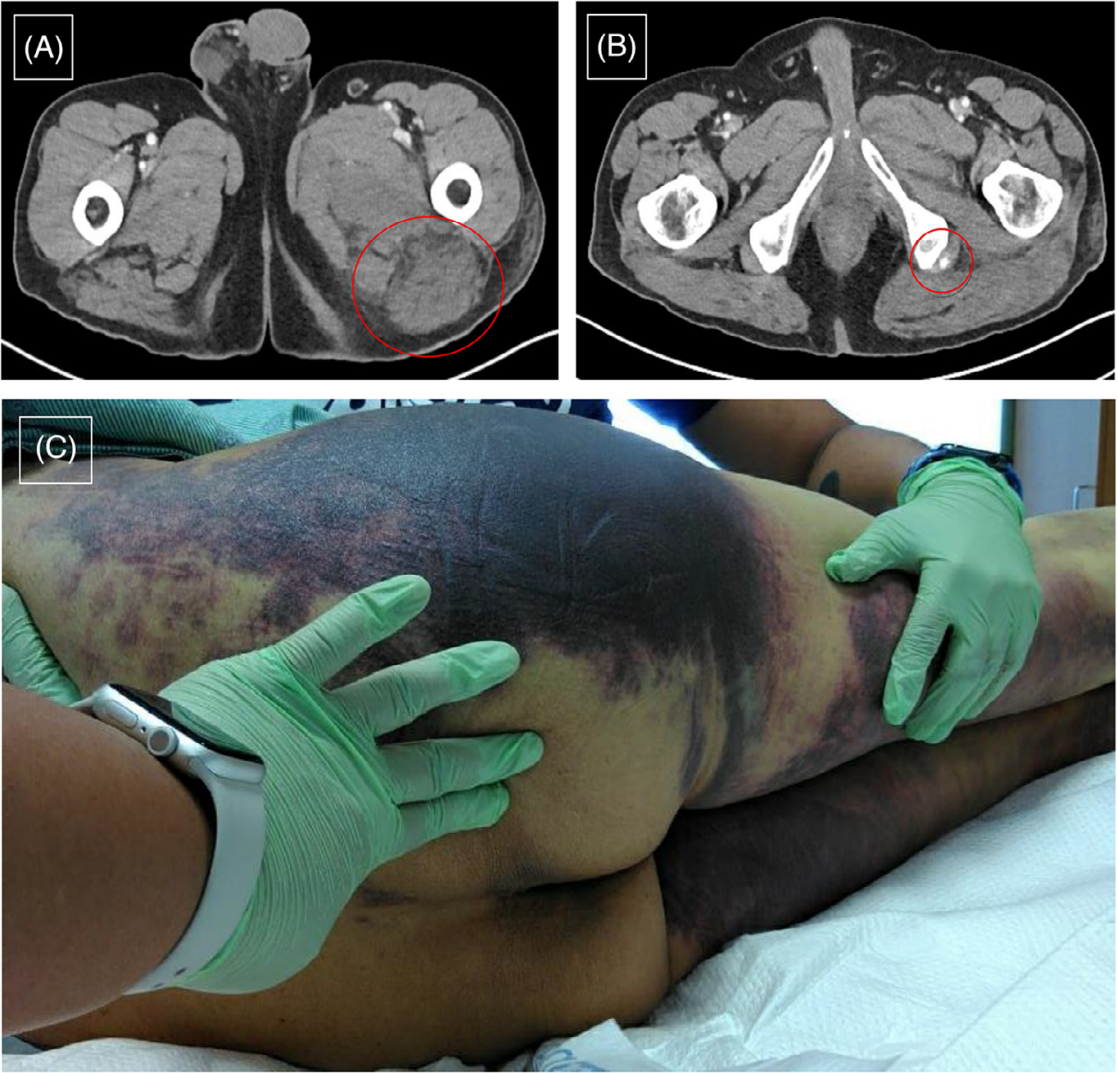
(A) Computed tomography (CT) abdomen/ pelvis axial section showing diffuse intramuscular hematoma formation throughout the posterior compartment of the left upper thigh. (B) CT axial section demonstrating active bleeding, likely from the left inferior gluteal vessels. (C) Physical examination showed extensive ecchymoses from retroperitoneal hematoma, tracking down the flank into the leg.

A 66‐year‐old Hispanic male with gout and hypertension presented with spontaneous bruising, inability to walk, and unintentional weight loss. He had no personal or family history of bleeding disorders, malignancy, or autoimmune disease. A physical exam showed ecchymosis along his right arm, tracking down to the flanks, thighs, and calves. Computed tomography (CT) imaging showed intramuscular hematoma in the posterior compartment of the left thigh and active bleeding from the left inferior gluteal vessels (Figure 1A,B). Activated partial thromboplastin time (APTT) was prolonged at 70.5 s (ref: 23.3–38.6 s) and did not correct with mixing study (53.8 s). FVIII activity was undetectable at < 1%; maximum FVIII inhibitor titer was 430 BU/mL (ref: < 0.6 BU/mL) using Nijmegen‐Bethesda assay.

### 2.2 Case 2

A 64‐year‐old Hispanic male with a past medical history of type 2 diabetes, chronic kidney disease, gout, and nonalcoholic steatohepatitis presented with calf pain after mild blunt trauma to the left leg. He denied any personal or family history of bleeding disorders or malignancy. On physical exam, he had significant ecchymoses on both lower extremities (Figure 1C). Hemoglobin was 5.2 g/dL with hypotension requiring vasopressor management. CT imaging demonstrated retroperitoneal hematoma tracking into the left thigh. APTT was prolonged at 52.2 s (ref: 23.3–38.6 s) and did not correct with mixing study (53.3 s). FVIII activity was undetectable at < 1% with maximum inhibitor titer 353 BU/mL.

### 2.3 Treatment and monitoring

Recombinant Factor VIIa 90 mcg/kg/dose was used every 2 to 4 h to stabilize bleeding; immunosuppression was initiated using prednisone 1 mg/kg daily and cyclophosphamide 100 mg daily, continued for six weeks, with rituximab 375 mg/m^2^ weekly for four doses. Patient 1 transitioned to rpFVIII while we investigated treatment on a clinical trial; unfortunately, due to location in El Paso, TX this was not possible. Both patients decided for off‐label treatment with emicizumab protocol of loading dose 3 mg/kg subcutaneously for 2–3 weekly doses followed by maintenance 1.5 mg/kg every 1 to 3 weeks adjusting frequency to maintain lowest effective FVIII activity with no breakthrough bleeding [9]. During maintenance, weekly monitoring was recommended with dosing frequency: administer dose of 1.5 mg/kg for FVIII < 10%, prolong the interval for FVIII activity 10%–30%, and hold dosing at higher levels.

After 3 weekly loading doses, Patient 1 began to walk independently with no new bruising and resolution of old bruising and resumed light work as an accountant. Patient 2 began emicizumab earlier without rpFVIII and rapidly improved clinically after 2 weekly loading doses. After 3 and 2 weeks respectively, both transitioned to maintenance treatment with monitoring. While on emicizumab, the Factor VIII activity chromogenic assay using bovine reagents was employed (normal range 50%–160%; Quest Diagnostics Nichols Institute, San Juan Capistrano, CA 92675). Emicizumab dose was adjusted for changes in weight of ±20%. Patients came to the infusion center with each treatment, obtaining clinical and laboratory monitoring with nursing staff, and hematology clinic follow up every 4–6 weeks.

### 2.4 Outcomes

**FIGURE 2:**
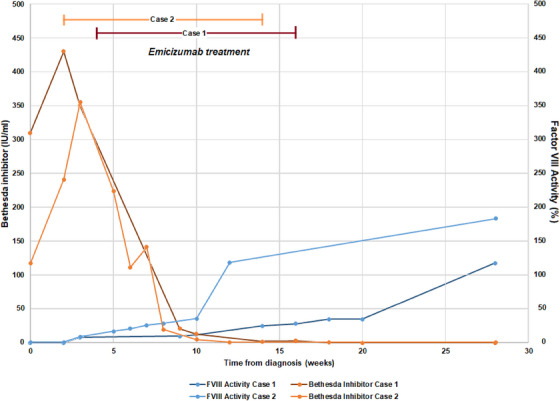
Factor VIII activity and inhibitor titers before, during, and after emicizumab treatment.

Both patients had steady recovery with no rebleeding, re‐hospitalization, and no adverse effects of any type. Figure 2 summarizes treatment courses. For Case 1, after emicizumab therapy for about 2 months, FVIII activity was 28% and FVIII inhibitor level 3 BU/mL. Case 1 discontinued emicizumab after 3 months therapy with FVIII activity 35% on chromogenic assay and undetectable inhibitor. For Case 2, after emicizumab therapy for about 2 months, FVIII activity was 21% and Factor VIII inhibitor level 19 BU/mL. Case 2 also stopped emicizumab at 3 months therapy with undetectable inhibitor and normal FVIII activity. At 1 year post‐diagnosis, both patients remain in complete remission and have resumed normal activities with no post‐treatment safety signals noted.

## 3 DISCUSSION

Management of severe AHA with high inhibitor titers is complex and anxiety‐provoking for patients and providers. Emicizumab is a bispecific recombinant monoclonal antibody containing two different antigen‐binding fragments [9]. One fragment recognizes Factor IXa and the other recognizes Factor X, resulting in spatial approximation and activation of Factor X and mimicking in vivo FVIII activity [10].^ ^With a long half‐life, emicizumab can be administered outpatient and has reduced the annualized bleeding rates in congenital hemophiliacs with and without inhibitors.

In 2021, Knoebl et al. reported emicizumab use for AHA in Austria [9]. Our approach followed this dosing regimen as detailed above with the exception that our patients were younger with good performance status and received concurrent intensive immunosuppression. While the cases presented above had higher maximum inhibitor titers, outcomes were similar, suggesting emicizumab maintains in vivo efficacy even in high FVIII inhibitor environments. This dosing regimen is limited by sample size but appears to have excellent hemostatic activity with clinical control of bleeding, no bleeding mortality and low thromboembolic rates (Table 1). Even low emicizumab plasma concentrations therefore appear to protect from bleeding [9].

**TABLE 1:** Summary of prospective studies using different emicizumab dosing and immunosuppression strategies for the treatment of acquired hemophilia A (AHA).

Study (*n*)	Median age (range), years	Associated disorders	Emicizumab dosing	Immunosuppressive therapy	Bleeding on treatment with emicizumab	Thromboembolic events	Mortality rate from bleeding
Austria: Knoebl et al., 2021 (*n *= 12)	74 (51–87)	One (8.3%) autoimmune disease; Three (25%) malignancy	Loading: 3 mg/kg subcutaneously, weekly for 2–3 doses Maintenance: 1.5 mg/kg every 3 weeks	Short course prednisone and/or rituximab^a^	0 (0%)	One (8.3%) stroke	0 (0%)
Japan: Shima et al., 2023 (*n *= 12)	76 (50–92)	Three (25.0%) autoimmune diseases and five (41.7%) malignancies.	Loading: 6 mg/kg on day 1 and 3 mg/kg on day 2 Maintenance: 1.5 mg/kg once weekly from day 8 onward	Prednisolone in all with cyclophosphamide in three (25%) and cyclosporine in one (8.3%)	5 (41.7%)	One (8.3%) deep vein thrombosis	0 (0%)
Germany and Austria: Tiede et al., 2023 (*n *= 47)	76 (21–93)	Seven (15%) autoimmune diseases, six (13%) malignancies, and one (2%) postpartum		None allowed for the first 11 weeks, then 29 (61.7%) received immunosuppression per investigator choice from week 12 or later	14 (29.8%)	One (2.1%) ischemic stroke.	2 (4.3%)

^a^ All patients in this study were reported to have had comorbidities that prevented intensive immunosuppressive therapy.

In 2023, two studies (AGEHA, Japan and GTH‐AHA‐EMI, Germany and Austria) were reported using emicizumab with limited or no immunosuppression and accelerated/modified emicizumab dosing regimen (Table 1) with loading on Days 1/2 and maintenance from Day 8 continuing weekly [11, 12]. This modified dosing was extrapolated from the phase III study of CHA with inhibitors (HAVEN1) which reported emicizumab efficacy was maximized at plasma concentrations > 30 μg/mL [13]. Outcomes of these 2 studies were promising with low bleeding mortality (0‐4%) and low thromboembolic event (2‐8%) rates. However, on‐treatment bleeding was still noted (Table 1). We look forward to the ongoing trial [NCT05345197] which will allow better comparison by reporting on accelerated/modified emicizumab dosing along with early immunosuppression [14].

## 4 CONCLUSION

These cases suggest that emicizumab with intensive immunosuppression is safe and efficacious in AHA patients with good performance status. The dosing regimen used had excellent hemostatic activity with clinical control of bleeding even in high inhibitor environments; it appears even low emicizumab plasma concentrations can protect from bleeding. The optimal dosing regimen remains to be defined from randomized prospective studies.

## CONFLICT OF INTEREST STATEMENT

The authors declare no conflict of interest.

## FUNDING INFORMATION

No sources of funding are applicable to this article.

## ETHICS STATEMENT

Data were collected in accordance with institutional protocols.

## PATIENT CONSENT STATEMENT

Patient consent was obtained and available on request.

## CLINICAL TRIAL REGISTRATION

Not applicable

## Data Availability

The data that support the findings of this study are available from the corresponding author upon reasonable request.
